# Characterization of *Salmonella* Dublin isolated from bovine and human hosts

**DOI:** 10.1186/s12866-019-1598-0

**Published:** 2019-10-16

**Authors:** Narayan Paudyal, Hang Pan, Mohammed Elbediwi, Xiao Zhou, Xianqi Peng, Xiaoliang Li, Weihuan Fang, Min Yue

**Affiliations:** 1 Institute of Preventive Veterinary Medicine, College of Animal Sciences, Zhejiang University, Hangzhou, China; 2 Animal Health Research Division (AHRD), Nepal Agricultural Research Council (NARC), Kathmandu, Nepal; 3 Zhejiang Provincial Laboratory of Preventive Veterinary Medicine, Hangzhou, China

**Keywords:** *Salmonella* Dublin, Antibiogram, Phenotype, Genotype, Morphotype, Virulence

## Abstract

**Background:**

*Salmonella enterica subsp. enterica* serovar Dublin (*S*. Dublin), a cattle adapted serovar causes enteritis, and systemic disease in bovines. The invasive index of this serovar far exceeds that of the other serovars and human infections often present as fatal or highly resistant infections. In this, observational study, phenotypic properties of human and bovine-derived isolates of *S*. Dublin along with antibiogram of common antimicrobials were evaluated. The multiplex PCR confirmed isolates were genotyped using 7-gene legacy MLST. MIC assay was done by broth microdilution method. Previously published protocols were used to assess the motility, biofilm formation and morphotype. Vi antigen was agglutinated using commercial antiserum. *Caenorhabditis elegans* infection model was used to evaluate the virulence potiential. Phenotyping experiments were done in duplicates while virulence assay was done in triplicates. Whole-genome sequencing was used to predict the genes responsible for acquired resistance and a genotype-phenotype comparison was made.

**Results:**

We evaluated 96 bovine and 10 human isolates in this study. All the isolates belonged to ST10 in eBG53 and were negative for Vi-antigen. The swarming motility, biofilm formation and morphotype were variable in the isolates of both groups. Resistance to sulfamethoxazole, ampicillin, chloramphenicol, tetracycline was > 90% in animal isolates whereas resistance to sulfamethoxazole was > 70% in human isolates. MDR was also higher in animal isolates. Human isolates were significantly (*P* < 0.0001) more virulent than animal isolates on *C. elegans* infection model. The genomic comparison based on the core SNPs showed a high degree of homogeneity between the isolates. The carriage of IncA/C2 plasmid was seen as a typical feature of isolates from the bovine hosts.

**Conclusion:**

Human isolates showed more diversity in the phenotypic assays. Animal isolates showed a higher degree of antimicrobial resistance with greater MDR but human isolates formed more biofilm and had greater swarming motility as well as increased virulence to the nematode *C. elegans*. The carriage of IncA/C2 plasmid could contribute to the distinguishing feature of the bovine isolates. The tandem use of genotypic-phenotypic assays improves the understanding of diversity and differential behaviour of the same serovar from unrelated host sources.

## Background

The non-typhoidal, *Salmonella enterica subsp. enterica* serovar Dublin (*S*. Dublin), cattle adapted serovar causes enteritis and/or systemic disease in bovine hosts [1]. It can also infect other animals including humans [2]. It causes invasive infections and fatalities in humans who have predisposing conditions like debility and chronic infections [3, 4]. An American study reported that the incidence rate for *S*. Dublin in humans increased by 7.6 times in 2013 as compared to the 1960s. The surge in multidrug resistance isolates was recorded in about 55% of the total isolated isolates [5]. Animal contact is the most frequently considered driver of pathogen dynamics, but a study from Denmark [6] suggested that proximity to cattle and the risk of infection are independent of each other. In China, Salmonella is one of the major foodborne bacteria [7, 8], *S*. Dublin is infrequently reported to cause foodborne outbreaks in humans [9]. This has also been isolated from blood and sputum [10], pediatric or infant patients [11, 12], and cases of the hepatic abscess [13, 14]. Like cattle, the high altitude yaks are also found to be positive for infections with this serovar [15].

Characteristics such as the capacity for biofilm formation, morphotype, motility, and antimicrobial resistance of pathogens, aids in successful colonization and persistence in hostile environments [16]. These properties could also contribute protective defence artillery of the pathogen against any incumbent intimidating situation. Earlier reports have shown the genotypic and/or phenotypic differences within the same serovar isolated from the different host [17, 18]. Based on these premises, we investigated the differences between the serovar Dublin from bovines and humans at genotypic as well as phenotypic level. The information available on the comparative phenotypic properties of this serovar isolated from different hosts is patchy. The animal isolates were collected over various years from different farms whereas human isolates were collected from hospitals.

## Results

Out of 108 isolates, 96 bovine and 10 human isolates were confirmed as *Salmonella* Dublin type by the multiplex PCR. Two human isolates did not show the necessary banding pattern after the mPCR. Whole-genome sequence analysis of these isolates in Enterobase showed that these non-Dublin serovars were serovars Javiana and Agona.

### MLST

The MLST was determined by using the sequences of seven housekeeping genes as described previously [19]. All the isolates belonged to sequence type 10 (ST10) in the e-burst group 53 (eBG53).

### Motility, biofilm formation, morphotype and Vi-antigen assay

**Fig. 1:**
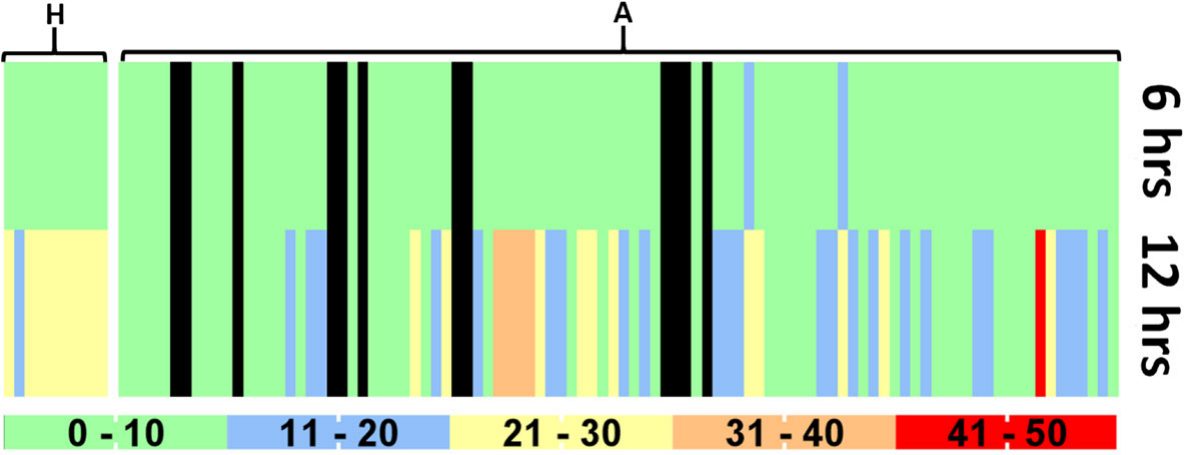
Comparative swarming motility of the isolates. H denotes the group of human (10) isolates, A denotes group of animal (96) isolates. The legend at the bottom categorizes the diameter of swarming motility (in mm) according to the colour at two-time points as denoted in the right. The black colour (not shown in the colour legend) denotes that these isolates did not show swarming motility

At the two time-points (six hours and 12 h of incubation), the difference in swarming motility was highly significant (*P* < 0.0001). The estimate of variance in motility as a factor of the individual isolate was 42.3% in animal isolates in contrast to 2.0% of human isolates. Similarly, the variance in motility as a factor of incubation time was 23.3% for animal isolates and 93.4% for human isolates. The comparative swarming motility of these isolates is presented as a heat map in Fig. 1.

On the evaluation of the biofilm formation assay, two animal isolates [2%], were classified as weak biofilm formers (the OD of the solution read at wavelength 492 in a spectrophotometer is smaller than 0.01). Three human isolates [30%] were biofilm formers out of which two were trace biofilm formers (the OD value of 492 is between 0.01 and 0.1, but no inclusion) while one was moderate biofilm former (OD492 is greater than 0.1). All other remaining isolates were non-biofilm formers.

**Fig. 2:**
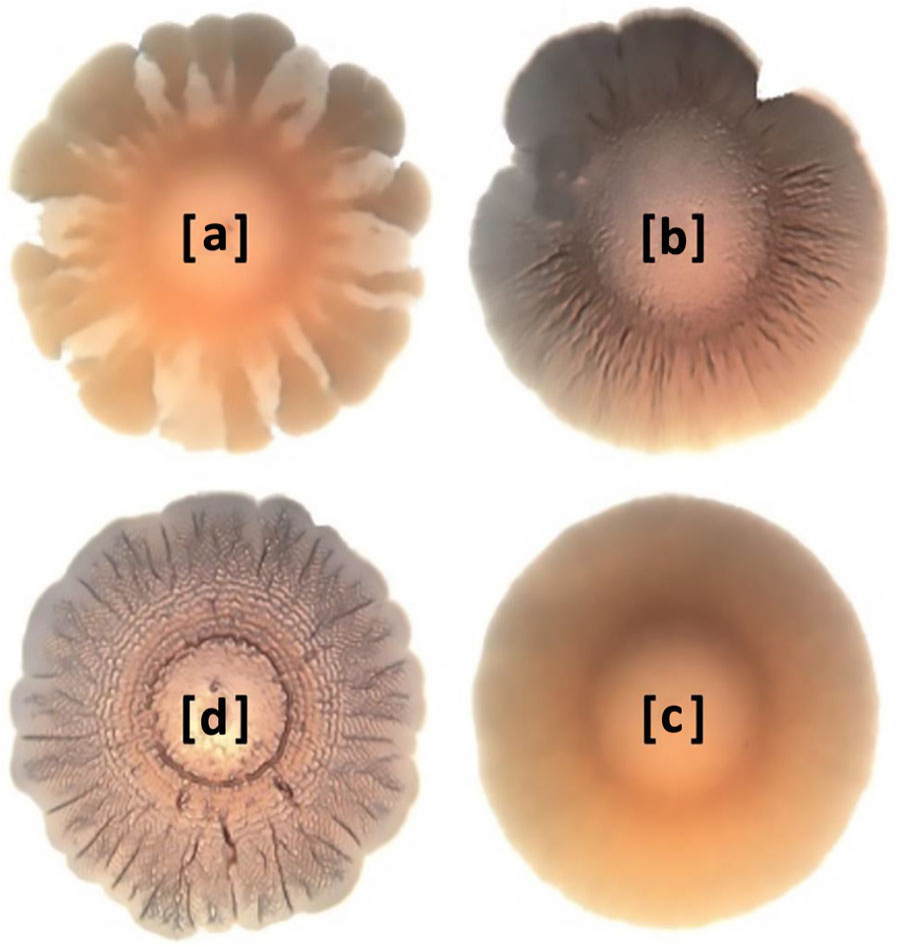
*S*. Dublin morphotype. **a**, **b** and **c** show the brown dry and rough (BDAR) or some of its variant morphotype that was recorded in most of the *S*. Dublin isolates used in this study, **d** shows the red, dry and rough (RDAR) morphotype recorded only in one animal and two human isolates

Regarding the morphotype, only one animal isolate and two human isolates formed the typical classic red, dry and rough (rdar) morphotype akin to *S*. Typhimurium. Rest of the isolates formed the brown, dry and rough (bdar) or some kind of its variant morphotype (Fig. 2). Only two human isolates formed both biofilm and rdar morphotype.

The agglutination test for Vi-antigen, done using a commercial antiserum did not show any positive agglutinations. All isolates were recorded as negative for Vi antigen.

### MIC assay

**Fig. 3:**
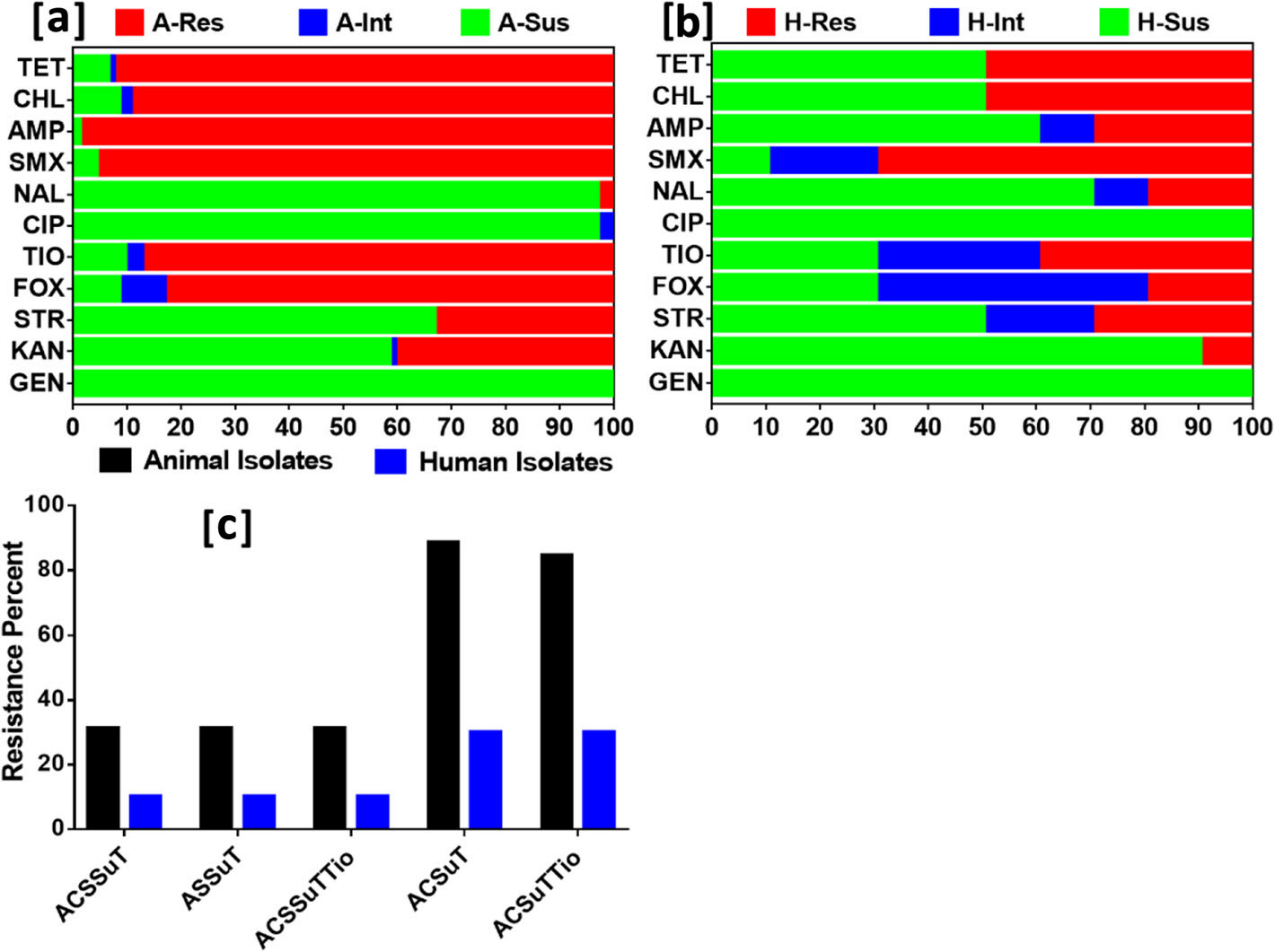
Antibiogram of the (**a**) Animal (**b**) Human isolates. Abbreviations are Res: resistance, Int: intermediate and Sus: susceptible. The XX’ has the units of per cent, YY’ represent the individual antimicrobials used which are abbreviated as GEN: gentamicin; KAN: kanamycin; STR: streptomycin; CIP: ciprofloxacin; NAL: nalidixic acid; TIO: ceftiofur; SMX: sulfamethoxazole; AMP: ampicillin; CHL: chloramphenicol and TET: tetracycline. **c** The tetra-, penta- or hexa- resistant patterns (percentage) of animal and human isolates. ASSuT (resistance to ampicillin, streptomycin, sulphamethoxazole and tetracycline except for the chloramphenicol); ACSSuT (resistance to ampicillin, chloramphenicol, streptomycin, sulphamethoxazole and tetracycline) or, ACSSuTTio (resistance to these aforementioned plus ceftiofur)

The results of micro-broth dilution assay showed that the overall resistance was higher in animal isolates than in human isolates. The percentage of bovine and human isolates, showing resistance to gentamicin and ciprofloxacin was similar, with distinct variations to tetracycline, chloramphenicol, ampicillin, sulfamethoxazole, ceftiofur and cefoxitin in which the resistance was higher in bovine isolates than in human isolates. In the bovine isolates, the highest resistance was towards tetracycline (92%), sulfamethoxazole (95%) and ampicillin (98%) (Fig. 3a). The human isolates were resistant to ceftiofur (40%), chloramphenicol, tetracycline (50%) and sulfamethoxazole (70%) (Fig. 3b). Human isolates were more frequently classified with intermediate resistance than the bovine isolates, with the highest being for the cephalosporins (30–50%). A higher rate of the tetra-, penta- and hexa- drug resistance patterns (31–88%) among the bovine isolates was calculated from the analysis (Fig. 3c).

### Genomic evaluation

The phenotypic resistance was compared with the acquired resistance genes analyzed from the ResFinder in CGE server. Concordance between the phenotypic and genotypic resistance was seen in > 80% of the isolates for the various antibiotics used. The least discordance among the animal isolates was seen for sulfamethoxazole (4.5% mismatch) while the most were seen for aminoglycosides (30% mismatch). In the human isolates, there was no mismatch for fenicols, quinolones and tetracyclines whereas it was 40% for sulfamethoxazole. The most common mismatch (dissimilarity between the genotypic and phenotypic results) was the genotypic presence of acquired resistance genes but the phenotypic absence of resistance (in aminoglycosides) and absence of genotypic resistance but the presence of phenotypic resistance (for beta-lactams).

Comparison of acquired resistance genes in animal isolates showed that twenty-one isolates shared three genes, *sul2*, *aph (6)-Id*, *aph (3″)-Ib* in common. Seventeen isolates shared two genes *floR* and *tetA* in common. *blaCMY-2* was common in 14 isolates, *blaTEM-1B* in three isolates, *aph (3′)-Ia* in one isolate and *blaTEM-116* in one isolate. In the five human isolates, the common among all was *aac (6″)-Iaa*, the same as that of the animal isolates. Only two isolates shared, *floR*, *tetA*, *sul2*, *aph (6)-Id* and *blaCMY-2* in common while only one isolate had the gene *blaTEM-1B*. No known point mutations leading to quinolone resistance was revealed in any of the isolates. Among the detected plasmids, 18/22 animal isolates and 2/5 human isolates harboured the IncA/C2 plasmid (100% identity), all harboured IncX1 (98.6% identity) and IncFII(S) (97.7% identity).

**Fig. 4:**
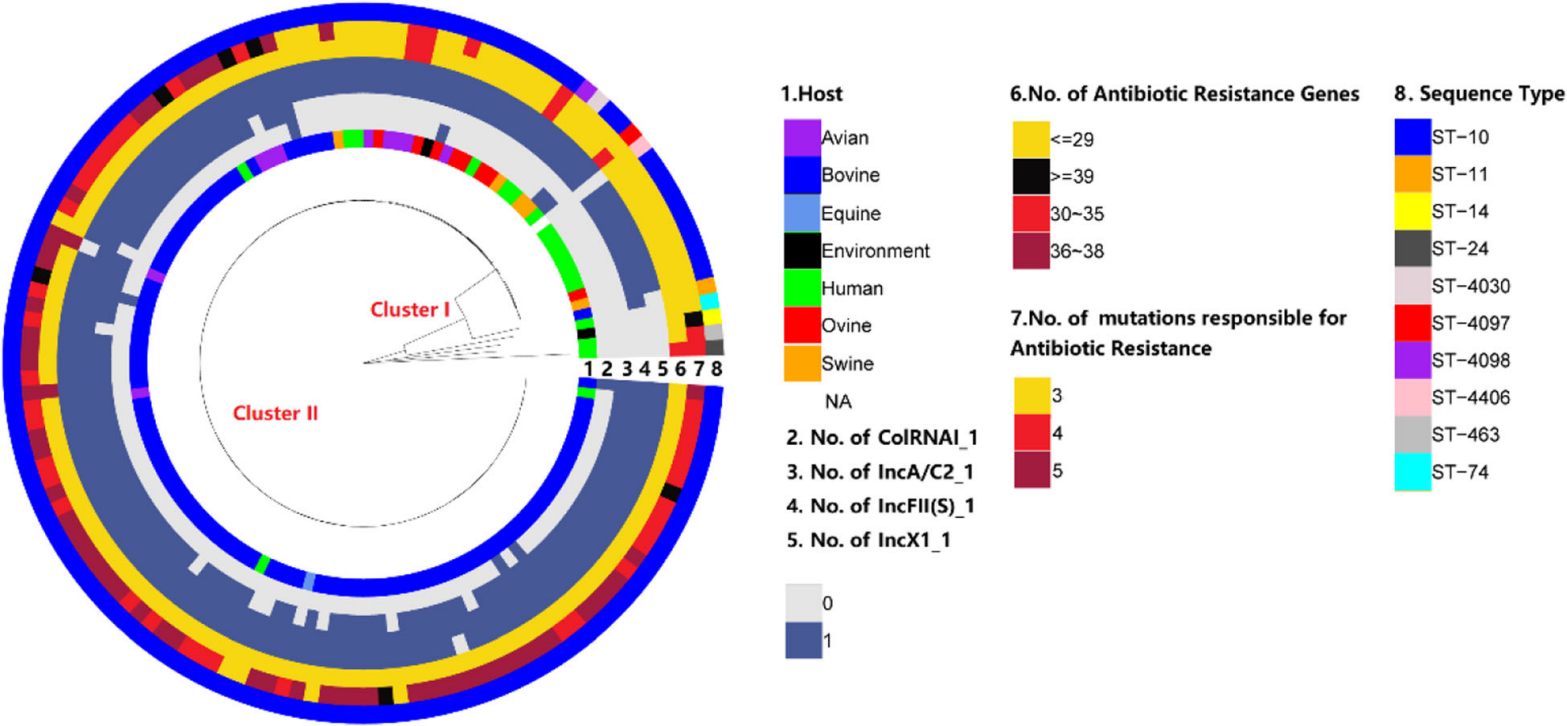
Genomic comparison of *S*. Dublin. Cluster I includes the isolates collected from other sources and reference strains (not included in our other laboratory assays) as well as our human isolates whereas the Cluster II includes all of our bovine strains. Each ring of the figure marked by numbers from one to eight represents eight different kinds of variables as indicated in the legend (on the right) and the colouring indicates the different sub-variables for each variable category. For the list of isolates and the variables, please refer to the Additional file 1

While all our bovine isolates aggregated in one segment [Cluster I], isolates from human were interspersed with those of other animals but separating away from the bovine isolates [Cluster II]. While the bovine and human isolates were distinct in terms of phylogeny, all the bovine isolates were in proximity to each other (Fig. 4).

### Virulence assay in *C. elegans*

The shortest median survival time of worms fed on the isolates of human origin was seven days while that for the worms fed on the animal isolates was nine days under identical conditions. The Gehan-Breslow-Wilcoxon analysis revealed that the survival curves among these isolates were significantly different (*P* < 0.0001).

## Discussions

*Salmonella* Dublin, a host-adapted serovar usually causes typhoidal disease in cattle and inflicts severe illness, along with severe bacteremia in humans [20]. It has a higher invasiveness and pathogenicity than other commonly encountered serovars [21, 22]. With the increase in the use of whole-genome sequencing in clinical human or veterinary microbiology, many researchers are moving away from the classical phenotypic evaluation of the pathogen [23]. However, such results of WGS must be interpreted with caution because the genomic presence/absence does not necessarily imply the clinical relevance [24].

Currently, ST10 is the major ST of serovar Dublin [1, 25]. More than 90% of this serovar deposited in the Enterobase belong to the ST10, eBG 53. The global *S. Dublin* is a highly homogenous population [26], even with the isolates that were collected in the past [27].

Property of motility that helps, to either invade or evade the infection site is a useful property during the colonization and pathogenicity [28]. The observed swarming motility was different among the isolates. The diameter of motility of human isolates was typically larger and more uniform than the diameter of motility of bovine isolates, which varied within a larger range. An earlier study suggests the absence of flagella in *S*. Dublin isolates [25], which corroborates the absence of motility in the animal isolates as seen from our assay. The swarming phenomenon is generally preceded by some differentiation of short swimmer cells into morphologically multinucleated and hyper-flagellated swarmer cells [29], which being a time-dependent variable relates to the influence of the incubation length on the motility. The animal isolates (more host-adapted) formed no biofilm (except one strain) as compared to the human isolates (more promiscuous). The biofilm formation ability is apparently reported to be positively correlated with the ability to infect and colonize multiple host species [30]. The human isolates that formed more biofilm showed rdar morphotype but the animal isolate did not. Host-adapted serovars such as Gallinarum, Cholerasuis and Typhi are sometimes reported to be entirely rdar negative [31] but none report about the serovar Dublin. Many isolates formed brown, dry and rough (bdar) or some of its variant morphotype despite which there was no biofilm formation in the majority of such isolates. Presence of curli which is important for biofilm formation in *S*. Typhimurium [32] did not necessarily confer biofilm formation in these isolates. The culture media potentially influenced biofilm formation. Among the gene cascade that controls biofilm formation in salmonellae, the adrA expression is very low in nutritionally rich media thus restricts the amount of biofilm formed [32].

Animal isolates were more resistant to tetracycline, chloramphenicol, ampicillin, sulfamethoxazole, and cephalosporins. These antibiotics represent the most common groups of antimicrobials used in commercial animal farming in China [33]. While we cannot exactly show cause-effect relationship due to the absence of relevant metadata and the roles of possible dark matters in the dynamics of antimicrobial resistance [34], the presence of a higher degree of resistance to antimicrobials commonly and frequently used in farm animals certainly raises the red flag. The resistance to sulfamethoxazole was the highest among the human isolates. The higher percentage of intermediate categorized isolates in cephems highlights the volatility of the state. Similar risks and patterns of resistance have been reported by some earlier publications [35, 36].

Resistance to nalidixic acid by human isolates is also an interesting revelation. Despite the absence of genetic resistance determinants or known point mutations, other cellular mechanisms that influence quinolone resistance, the activation of efflux pumps or metabolic transformation could have come into play [37]. Quinolones are currently preferred as the first choice of drugs for the treatment of invasive enteric salmonellosis [38] and this could unwittingly be encouraging the intermediate state as seen in our analysis. The presence of higher rates of MDR in animals as compared to human isolates also corroborates the theory of multiple antibiotic usages in animal husbandry. The presence of a resistance gene does not necessarily confer phenotypic resistance, and the absence of resistance genes does not suggest the phenotypic susceptibility [24]. The phenomenon of AMR is not just related to the mere presence or absence of resistance genes. Other mechanisms such as enzyme activation, target modification/protection, regulation of AMR gene expression, or even change in the cell wall charge play some important roles in the AMR. So when compared with just the AMR genes, some degree of discordance is inevitable. Because of such multiple variables and manifold association of genotypic and phenotypic data [39], a comparison of genotype-phenotype should give a better and complete picture.

The *C. elegans* assay showed that human isolates were more virulent than the animal isolates. The human isolates rapidly killed most of the worms in the first 3–8 days of infection after which only a few worms remained alive for the protracted period. Animal isolates, on the other hand, killed a few worms every day over a long time. The immune mechanism of the worms could have overcome the virulence of the pathogen in those remaining alive, thereby increasing the survival days. The virulence trait of *S*. Dublin is likely to be a factor of host-pathogen interaction rather than the factor of the pathogen alone [40]. It is seen from Fig. 4, that the bovine isolates are relatively recent in the evolutionary process. It can be inferred from the phylogenetic tree that S. Dublin had earlier adapted to the humans and other animal hosts (probably the ovine) and then jumped to the bovine host in the latter period of evolution. The increasing interaction of the human and animals’ population thus providing optimized routes for zoonotic exchange could have driven this diversification on the SNPs recently. The strains in these clusters I and II not only differ in terms of their isolation source or host predilection but also in plasmid carriage and the number of acquired antibiotic resistance determinants. Isolates in Cluster I contained strains from humans, ovine, and environment that differed in the sequence types. Isolates of this cluster harboured either none or only two plasmids, namely, IncFII(S)_1 or IncX1_1. These isolates contained less than 29 acquired antibiotic resistance determinants and three mutations responsible for antibiotic resistance. Isolates in Cluster II were namely of the ST 10 from the bovine origin with very few avian (namely poultry) isolates intermixed. These isolates contained up to four different types of plasmids but the number of antibiotic resistance determinants was similar (< 29) to the isolates in Cluster I. The mutations responsible for antibiotic resistance ranged between four and five in all these isolates.

The motility, ability of biofilm production, and virulence capability assist the host-adapted serovars such as Dublin to successfully colonize the hosts. The absence of biofilm, but the presence of adequate swarming motility would allow the pathogen to move away from a hostile environment to a friendly niche. Simultaneously, the increase in resistance would greatly enhance the fitness of survival of the pathogen in the human/animal gut under the selective pressure of antibiotics use. It is possible that these differential properties are the factors of the host-pathogen interaction rather than the genomic composition of the pathogen alone. Despite the genomic similarity, the pathogen could diversify its interaction in multiple hosts thus elevating its probabilities of survival.

## Conclusion

This study reports antibiogram and characterizes the genotype-phenotype homogeneity and variability of the *S*. Dublin of animal and human origin. Genomically, the isolates were largely homogenous. However, on phenotyping, human isolates behaved distinctly and differently. Animal isolates had a higher antimicrobial resistance with greater MDR but human isolates formed more biofilm and had greater swarming motility as well as more virulence to the nematode *C. elegans*. The tandem use of genotypic-phenotypic assays can greatly improve our understanding of diversity and differential behaviour of the same serovar from different host sources.

## Methods

### Isolate collection, identification

A set of 108 salmonellae collected from multiple sources and stored at our laboratory was used in this study. Among these, 96 were the bovine isolates collected from dairy farms over various years (2007 to 2012) while 12 were the isolates collected from clinically sick humans by multiple hospitals around Zhejiang, Shenzhen and Shanghai (2011 to 2017). The bovine samples were collected during the regular epidemiological surveillance of animal disease, and from veterinary clinics at different geographical regions in the east coast, so there was no overlap between the humans and bovine isolates. No human patient personal data was available to the authors’ so informed consent or approval was not deemed necessary for the use of those isolates. The bovine isolates were identified to the genus level in the past by classical microbiological procedures whereas the human isolates were identified to the serovar level. All these isolates were re-confirmed to be true to the type using a specific multiplex PCR as recommended [41]. The genome for the downstream application was extracted using a commercial bacterial genome extraction kit (Tiangen Biotech, Beijing) and quantified using Nanodrop1000 (Thermo Fischer).

### Genotypic and phenotypic assays

Multilocus sequence typing using the seven housekeeping genes was done as recommended [19]. Tests for the evaluation of phenotypic properties like biofilm formation assay [42], morphotype assay [43], motility assay [44], and MIC (minimum inhibitory concentration) assay of antimicrobial agents [45, 46] were performed as described in the published literature. Agglutination for Vi-antigen was done using commercial antiserum. All the assays were done in duplicates. Virulence assay on nematode *C. elegans* was done in triplicates [47].

### Genomic analysis

All the 106 isolates were whole-genome sequenced using Illumina Hiseq Platform by a commercial vendor. The raw reads were quality checked and assembled in the Galaxy Platform [48]. The assembled contigs in FASTA format were annotated in the RAST vs 2 [49]. The annotated contigs were analyzed for acquired resistance genes, point mutations and plasmids using CGE PlasmidFinder vs 2.0 [50] and ResFinder vs 3.1 [51]. The details of the various parameters that were obtained from the genomic analysis are given as the Additional file 1.

In addition to our 106 strains of S. Dublin, 34 more were downloaded from the Enterobase and one reference strain from the NCBI, to make a heterogeneous population. The sequences downloaded were those of the isolates from different hosts at multiple countries for non-related periods. The assembled contigs were submitted to the CGE server to analyse the overall distribution of the acquired antimicrobial resistance determinants and the plasmids. The contigs were used for variant calling against reference strains ATCC 39184 by software Snippy 4.3.6 to obtain Core SNPs. After being filtered by 95% gap parameter to get the core SNPs, 92944 SNPs were used to build up a tree by IQtree (1.6.8), with the best model TVM + F + ASC. The same method was conveyed for building tree for Cluster I method (Total SNPs = 20688, No. of Core SNPs = 20688). Tree and metadata including MLST, AR genes, AR mutations, plasmids were combined by strain name column (as an index) and analyzed in R studio with R package, ggtree, ggplot2, data.table, treeio, miscTools, gridExtra, xlsx, phytools, phangorn, tidyverse, pheatmap and gheatmap to output a complete comparative circular illustration rooted by non-Dublin strain (serovar Javiana) and reference strain *S*. Dublin ATCC 39184, respectively.

Concordance (or discordance) was calculated as a percentage of the number of similar (or dissimilar) isolates obtained when the genotypic presence (or absence) of acquired resistance genes was compared to the phenotypic presence (or absence) of the acquired resistance determinants to that particular class of antibiotic agent.

### Data analysis

MLST data were analyzed in Enterobase. The biofilm formation data were analyzed as recommended in an earlier publication [42]. Survival data of *C. elegans* were analyzed with Kaplan Meier’s estimator. All numerical data were analyzed in GraphPad Prism vs 7 on Windows machine.

## Supplementary information

**Additional file 1.** Genomic Compare of S. Dublin. This is a spreadsheet elaborating the details like presence or absence of various acquired antibiotic resistance determinants, mutations, and plasmids. The first column (Column A) states the source of the isolates (our library strains-animal or humans) and the alien strains downloaded from the Enterobase database. Column B gives the strain identifier (name), Colum C gives the result of legacy 7 gene Salmonella MLST as calculated from the Enterobase algorithm, Column D gives the source of the isolate. Column E to Column FD elaborates the variables such as acquired antibiotic resistance determinants, mutations, Plasmids where 0 indicates the absence and 1 indicates the presence of that particular variable type.

## Data Availability

The datasets used and/or analyzed during the current study are available from the corresponding author on reasonable request.

## Abbreviations

AMR: Anti-Microbial Resistance
CGE: Center for Genomic Epidemiology
eBG: e-Burst Group
MDR: Multi Drug-Resistant
MIC: Minimum Inhibitory Concentration
MLST: Multi Locus Sequence Typing
mPCR: multiplex Polymerase Chain Reaction
ST: Sequence Type
WGS: Whole Genome Sequencing
